# Brain-scale cortico-cortical functional connectivity in the delta-theta band is a robust signature of conscious states: an intracranial and scalp EEG study

**DOI:** 10.1038/s41598-020-70447-7

**Published:** 2020-08-20

**Authors:** Pierre Bourdillon, Bertrand Hermann, Marc Guénot, Hélène Bastuji, Jean Isnard, Jean-Rémi King, Jacobo Sitt, Lionel Naccache

**Affiliations:** 1 Department of Neurophysiology, Hospital for Neurology and Neurosurgery, Hospices Civils de Lyon, Lyon, France; 2 Faculté de médecine Claude Bernard, Université de Lyon, Lyon, France; 3 Brain and Spine Institue, INSERM U1127, CNRS 7225, 47 boulevard de l’Hôpital, 75013 Paris, France; 4 Sorbonne Université, Paris, France; 5 Neuro Intensive Care Unit, Groupe Hospitalier Pitié-Salpêtrière, Assistance Publique Hôpitaux de Paris, Paris, France; 6 Neuropain Team, Centre de Recherche en Neurosciences de Lyon, INSERM U1028, Lyon, France; 7 Functional Neurology Department and Sleep Center, Hospices Civils de Lyon, Lyon, France; 8 Department of Neurophysiology, Groupe Hospitalier Pitié-Salpêtrière, Assistance Publique Hôpitaux de Paris, Paris, France

**Keywords:** Cognitive neuroscience, Computational neuroscience

## Abstract

Long-range cortico-cortical functional connectivity has long been theorized to be necessary for conscious states. In the present work, we estimate long-range cortical connectivity in a series of intracranial and scalp EEG recordings experiments. In the two first experiments intracranial-EEG (iEEG) was recorded during four distinct states within the same individuals: conscious wakefulness (CW), rapid-eye-movement sleep (REM), stable periods of slow-wave sleep (SWS) and deep propofol anaesthesia (PA). We estimated functional connectivity using the following two methods: weighted Symbolic-Mutual-Information (wSMI) and phase-locked value (PLV). Our results showed that long-range functional connectivity in the delta-theta frequency band specifically discriminated CW and REM from SWS and PA. In the third experiment, we generalized this original finding on a large cohort of brain-injured patients. FC in the delta-theta band was significantly higher in patients being in a minimally conscious state (MCS) than in those being in a vegetative state (or unresponsive wakefulness syndrome). Taken together the present results suggest that FC of cortical activity in this slow frequency band is a new and robust signature of conscious states.

## Introduction

Identifying a robust neural signature of conscious states constitutes a major scientific and medical challenge. While several theoretical and empirical works put forward the importance of cortico-cortical connectivity for conscious states^1–5^, this property remains highly debated regarding: (i) the long-range^6^ versus short-range type of functional connectivity (FC)^7^ (ii) the relevant frequencies involved in this cortico-cortical connectivity, with contradicting proposals ranging from ultra-slow (< 0.01 Hz)^8^, to slow (0.5–4 Hz)^9,10^, or even faster theta, alpha and gamma-band rhythms^11^, and (iii) the general value of this property when comparing various conscious and unconscious states.

**Figure 1:**
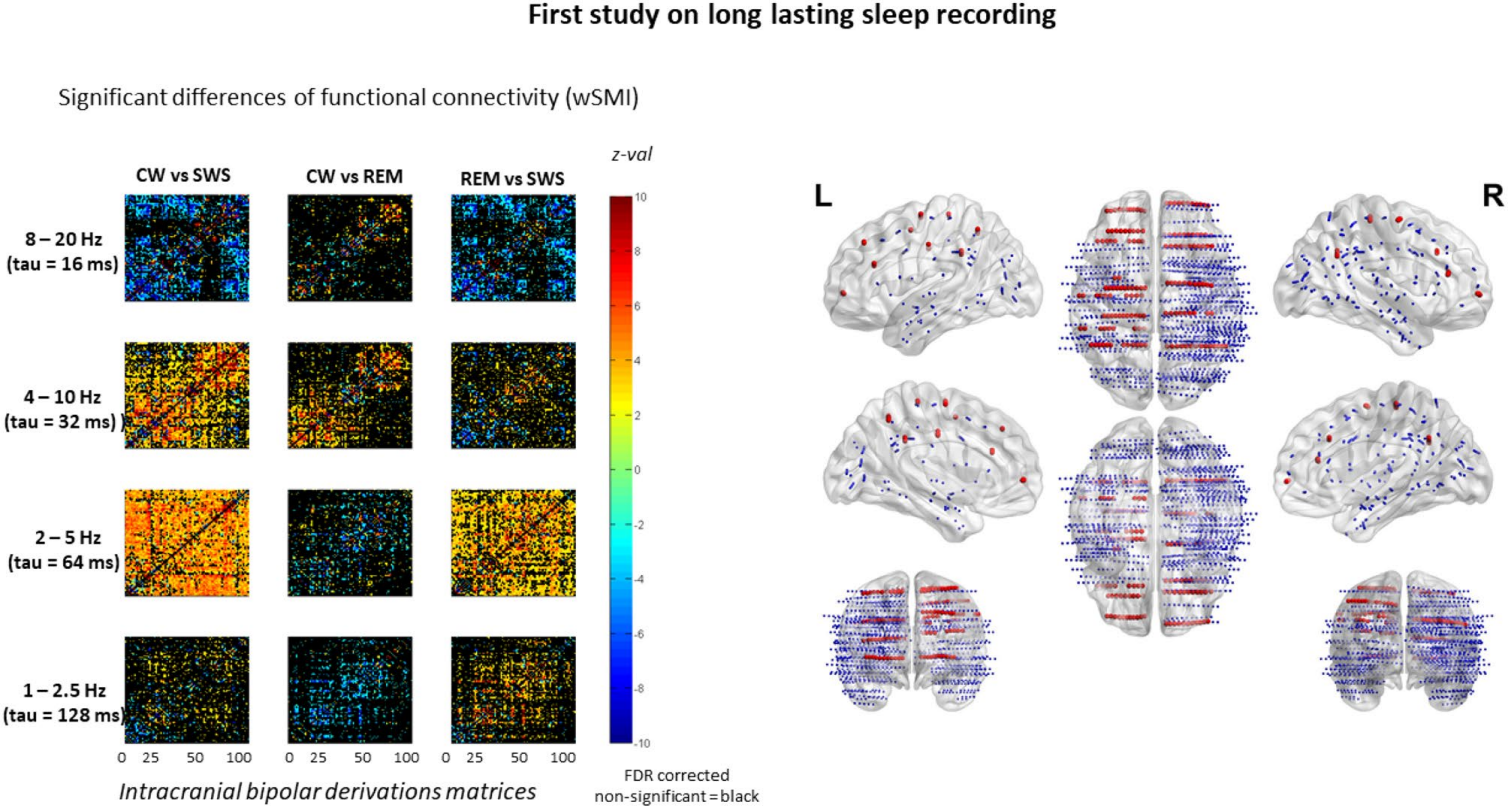
Matrices (bipolar derivations × bipolar derivations) of statistical comparison of the mean wSMI values between stages in patient 1. Color bar corresponds to the z-values. Non-significant results are plotted in black. Tridimensional representation of the electrodes in the MNI space. Electrode of patient 1 are plotted in red. *CW* conscious wakefulness, *SWS* N3 slow wave sleep, *REM* rapid eye movement sleep, *wSMI* weighted symbolic mutual information.

**Figure 2:**
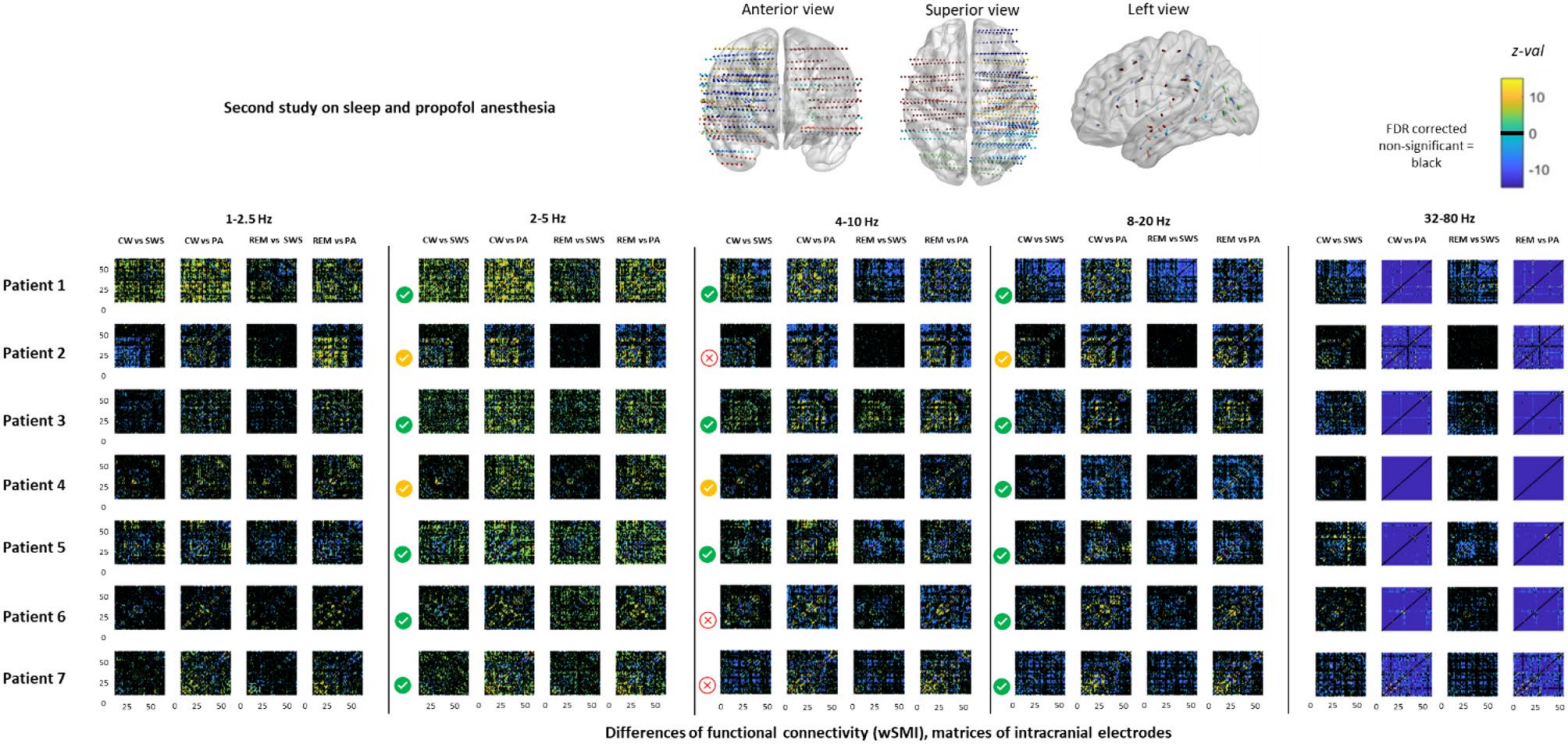
Individual anatomical data and functional connectivity matrices. On the top: Representation in the MNI space of all electrodes implanted in the 7 patients recorded in the four distinct stages (including PA). Each patient is associated to a single colour. On the bottom: Matrices (bipolar derivation × bipolar derivation) showing the z-values of the statistical comparisons between each bipolar derivation across the different stages for three ranges of frequencies: 2–5 Hz; 4–10 Hz, 8–20 Hz; 32–80 Hz. Only significant FDR-corrected z-values are represented. Non-significant effects are plotted in black. *CW* conscious wakefulness, *SWS* N3 Slow Wave Sleep, *REM* rapid eye movement sleep, *PA* propofol induced general anaesthesia. Symbols show which patients did statistically show the expected pattern in the three frequency bands highlighted by the first experiment:  = expected pattern;  = expected pattern but with one non-significant comparison;  = incongruent to the expected pattern.

In order to address these important and unsolved questions we first specified a clear definition of what we consider as a conscious state: a state during which experiences can be self-reported and memorized in episodic memory^12^. In that perspective, dreams that can be recalled as subjective self-reports belong to the class of conscious experiences. Given that most vivid and memorized dreams occur during REM-sleep or during transitions from SWS to REM-sleep^13–15^, this stage can be defined as a state that includes conscious episodes. Then, we explored cortico-cortical connectivity on direct intracranial EEG (iEEG) recordings performed in the same subjects across various conscious and unconscious states. Specifically, we recorded iEEG in 12 drug-resistant epileptic patients undergoing a stereoelectroencephalography (SEEG) for pre-surgical evaluation with brain-scale implantations (Figs. 1 and 2). iEEG recording was analysed and only the time-epochs free from epileptic abnormalities were used. We selected two states that are consensually considered as unconscious states during which there is no first-person subjective experience that can be self-reported: stable periods of stage 3 slow-wave sleep (SWS)^14^, and deep propofol anesthesia (PA). For conscious states, we selected stable periods of conscious wakefulness (CW), as well as stable periods of rapid-eye movement sleep (REM). Considering REM as a conscious state as we mentioned above deserves to be further developed and justified: first, since its discovery by Jouvet^16^, REM has been described as a state of fast, desynchronized and low voltage EEG resembling CW. This mismatch between an awake cortex and a sleepy appearance inspired the seminal name of REM that was “paradoxical sleep”. Second, awakenings studies revealed that most vivid, rich, detailed and subjectively reportable dreams do occur during REM. As such, this state therefore includes univocal episodes of conscious subjective experience that can be transferred to episodic memory. Recent works on REM sleep behavioural disorders (RBD),—allowing the recording of intentional motor behaviors associated to dream conscious contents (e.g.: smoking a cigarette; a military officer reviewing his troops)—, further confirmed that such consciously reportable dreams do occur during REM^13^. In contrast numerous studies confirmed that much less rich and vivid dreams seem to occur during SWS, and in particular during stable periods of stage 3 SWS^14^. We excluded transitional periods between SWS and REM, as well as stage 2 SWS that have been associated with some sparse and elementary dream experience^14^. Another argument supporting our approach originates from a series of TMS-EEG studies by Massimini and colleagues^17–19^. They reliably demonstrated that after a focal single pulse of TMS delivered to a focal cortical region elicited an early, focal and transient response both in CW and in various unconscious states including deep general anesthesia with several drugs, SWS, VS/UWS and comatose state, whereas only CW was associated with later cortical response. This late cortical response was distant from the initial focus of TMS including prefrontal and parietal associative areas, it was also sustained in time and complex. They could define a threshold value coined the perturbational complexity index (PCI) that distinguishes conscious from unconscious states. Crucially, REM was associated with high PCI values, undistinguishable from CW values. Taken together, all these arguments explain why, in the present study, we decided to group together CW and REM as conscious states.

Functional connectivity of scalp EEG, of magnetoencephalography (MEG) and of iEEG signals can be estimated and quantified through different mathematical measures. Among them, spectral and information theory methods have been developed^20–28^ to overcome the instantaneous propagation of electric fields generated by a primary current source to multiple sensors, which induce couplings that do not reflect true brain inter-regional interactions^29–31^. Of special interest, weighted Phase Lag Index (wPLI) and weighted Symbolic Mutual Information (wSMI) recently emerged as functional connectivity methods robust to artefactual coupling. While wPLI functional connectivity is sensitive to both linear and nonlinear interdependencies, weighted Symbolic Mutual Information (wSMI) (information-theoretic connectivity, see below) can detect purely nonlinear interaction dynamics^31^. King and colleagues conceived wSMI, and used this measure to distinguish conscious and minimally conscious (MCS) patients from vegetative state/unresponsive wakefulness syndrome (VS/UWS) patients^20^. wSMI evaluates the extent to which two EEG signals present non-random linear or non-linear joint-fluctuations. This method probes information patterns shared across two signals (see “Materials and methods”). The symbolic transformation depends on the number of time points that are used to define the symbol (k) and on the temporal separation between these time-points (sampling of EEG values each τ ms) enabling to probe various frequency bands. wSMI recently revealed an increase of cortical functional connectivity after the restoration of consciousness by vagus nerve stimulation in one patient in the VS/UWS^32^. Both King et al. study and this last study showed that MCS could be distinguished from VS/UWS with the wMSI computed in the 4–10 Hz (τ = 32 ms), while wSMI computed in higher frequencies failed to do so. In the present work, we capitalized on these previous findings and on the arguments listed above to choose wSMI to estimate functional connectivity. In order to reinforce the reliability of our findings, we complemented this approach by replicated our results with the the Phase Link Value (PLV)^33^ that is another and more extensively method used to quantify FC. In this study, tailored frequency bands have been chosen independently from the canonical frequency bands currently used in human electrophysiology. This choice was motivated by the previous publications on which the scientific hypothesis of the present study has been built^20,32,34^. In these publications, this tailored frequency band have been highlighted as crucial to correctly differentiate the different types of disorders of consciousness. Our goal was to test this neural signature in other conscious and unconscious states that the ones explored in these previous studies. Consequently we chose to use the same frequency band.

We conducted two consecutive SEEG studies: in Study I, five patients were recorded during 4 nights in three stages (CW, REM, SWS), whereas in Study II, seven additional patients were recorded during short periods of 10 min in four stages (CW, REM, SWS and PA). Finally, we conducted a third study using high-density scalp EEG on 145 patients suffering from disorders of consciousness (MCS and VS/UWS)^35^, in order to determine if our findings would discriminate between these two states.

## Results

### iEEG studies—conscious wakefulness, sleep and propofol anaesthesia

A total of 1777 recoding contacts (respectively 881 in the first study and 896 in the second) were implanted through 137 electrodes (respectively 67 in the first study and 70 in the second) covering the whole telencephalon (see Figs. 1 and 2). None of the 12 patients had any sleep disorder history. No modification of anti-epileptic drugs occurred during or between the recordings (see Table 1).

**Table 1:** Anti-epileptic drugs and location of epileptic foci of the patients of the two first studies.

First study	1st anti-epileptic drugs	2nd anti-epileptic drugs	Location of the epileptic focus
Patient 1	None	None	Frontal
Patient 2	Levetiracetam 2000 mg	Lamotrigine 800 mg	Frontal
Patient 3	Levetiracetam 1000 mg	Lamotrigine 800 mg	Insular
Patient 4	Carbamazepine 600 mg	Pregabaline 75 mg	Parietal
Patient 5	Carbamazepine 800 mg	Valproate 500 mg	Temporal

Second study	1st anti-epileptic drugs	2nd anti-epileptic drugs	Location of the epileptic focus
Patient 1	Lamotrigine 800 mg	Perampanel 12 mg	Frontal
Patient 2	Levetiracetam 1000 mg	Lamotrigine 800 mg	Insular
Patient 3	Pregabaline 75 mg	Carbamazepine 800 mg	Frontal
Patient 4	Valproate 500 mg	Carbamazepine 600 mg	Temporal
Patient 5	Levetiracetam 2000 mg	None	Occipital
Patient 6	Carbamazepine 1200 mg	Zonizamide 500 mg	Frontal
Patient 7	Lamotrigine 800 mg	Aucun	Temporal

Our previously published studies highlighted the importance of the FC in the 4–10 Hz frequency band to discriminate distinct states of consciousness impairments in patients suffering from disorders of consciousness (DOC)^20,21,35,36^. Consequently, we first computed wSMI in this frequency band in long-lasting iEEG sleep recordings (first SEEG study), and then replicated our results while computing it also under propofol anaesthesia (second SEEG study). In line with our original study, wSMI computed in the 4–10 Hz (τ = 32 ms) succeeded to distinguish CW from SWS in all patients in whom long lasting sleep recording was analysed during the first study: mean wSMI 4–10 Hz computed across all bipolar contacts combinations was larger during CW than during SWS (t-test p < 0.001 for each patient). Interestingly, the FC in REM had a level between these two other stages, significantly lower than during CW and higher than during SWS (t-test p < 0.001) (see Fig. 1).

We conducted our second study on additional patients by using shorter recordings (10 min vs 4 nights) and adding a propofol general anaesthesia condition. It is worth noticing that the very different duration of the recordings between these two studies make it difficult to proceed to a pooled statistical analysis of the identical experimental conditions. In this second study, we failed to replicate this result: while 4/7 patients did show the expected pattern, three of them showed a reverse pattern (larger mean wSMI 4–10 Hz values during SWS than during CW; p < 0.001), and one patient did not show significant difference between these two states. More surprisingly, wSMI in the 4–10 Hz frequency band failed to discriminate robustly other conscious (CW and REM) from unconscious states (selected very stable and sustained samples of SWS and PA). Indeed, only 3 out of 7 patients showed a significant larger mean wSMI 4–10 Hz for CW than PA, whereas the four other patients showed the reverse pattern with higher mean wSMI 4–10 Hz for PA. Similarly, wSMI 4–10 Hz was higher during REM than during SWS in only 5 out of 12 patients, while the 7 remaining patients showed an opposite significant pattern. Finally, only 2 out of 7 patients showed larger wSMI 4–10 Hz in REM than in PA, while the five remaining ones also showed the reverse pattern (see Fig. 2).

**Figure 3:**
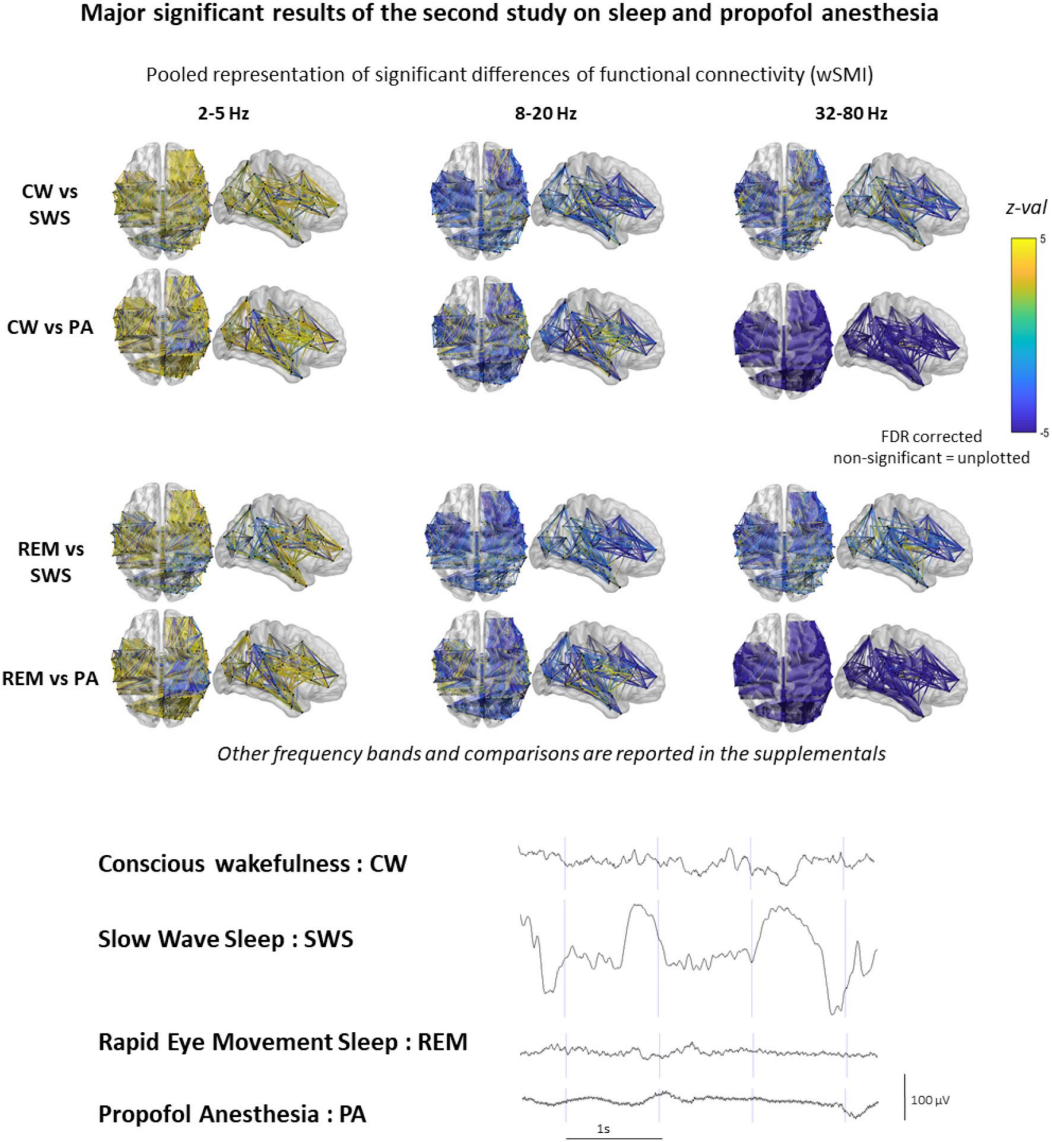
Anatomical representation of significant differences (z-values) of wSMI between stages in 2–5 Hz, 8–20 Hz and 32–80 Hz frequency bands. z-values of non-significant differences (after FDR correction) are not plotted. Data from the 7 patients are represented in a common MNI space. On the bottom, example of raw iEEG signal of each of the studied stages in the two first experiment. *UWS* unresponsive wakefulness state, *VS/UWS* vegetative state/unresponsive wakefulness syndrome, *MCS* minimally conscious state, *wSMI* weighted symbolic mutual information.

wSMI calculated on slower frequencies (wSMI 2–5 Hz; τ = 64 ms) appeared to better to discriminate the two conscious states from the two unconscious ones: 10 out of 12 patients showed significant larger mean values (*p* < 0.001) during CW than during SWS, while the remaining two patients (who both had short recordings during the second study) did not show significant differences across these two states. The very same result was found when comparing REM to SWS. In the 8–20 Hz (τ = 16 ms) and in the 32–80 Hz (τ = 4 ms), wSMI was significantly larger (*p* < 0.001) in the SWS as compared to CW and REM in all patients (Fig. 3). Finally, all patients had a larger wSMI in the 2–5 Hz frequency band during CW than PA, and during REM than PA (see Fig. 3). We then checked that wSMI computed in a slower frequency band (1–2.5 Hz; τ = 128 ms) and observed that it did not perform well to discriminate conscious from unconscious states (see Fig. 2).

We also noticed that PA, unlike SWS, was associated with a massive, diffuse and systematic increase of functional connectivity in high frequencies (32–80 Hz (τ = 2 ms), see Figs. 2 and 3) as compared to the three other states. For each comparison and each patient, more than 90% of the pairs of electrodes showed this effect. These findings are congruent with the increase of gamma band coupling recently described in non-human primates on electrocorticography during experimental anesthesia with propofol and ketamine^37^.

**Figure 4:**
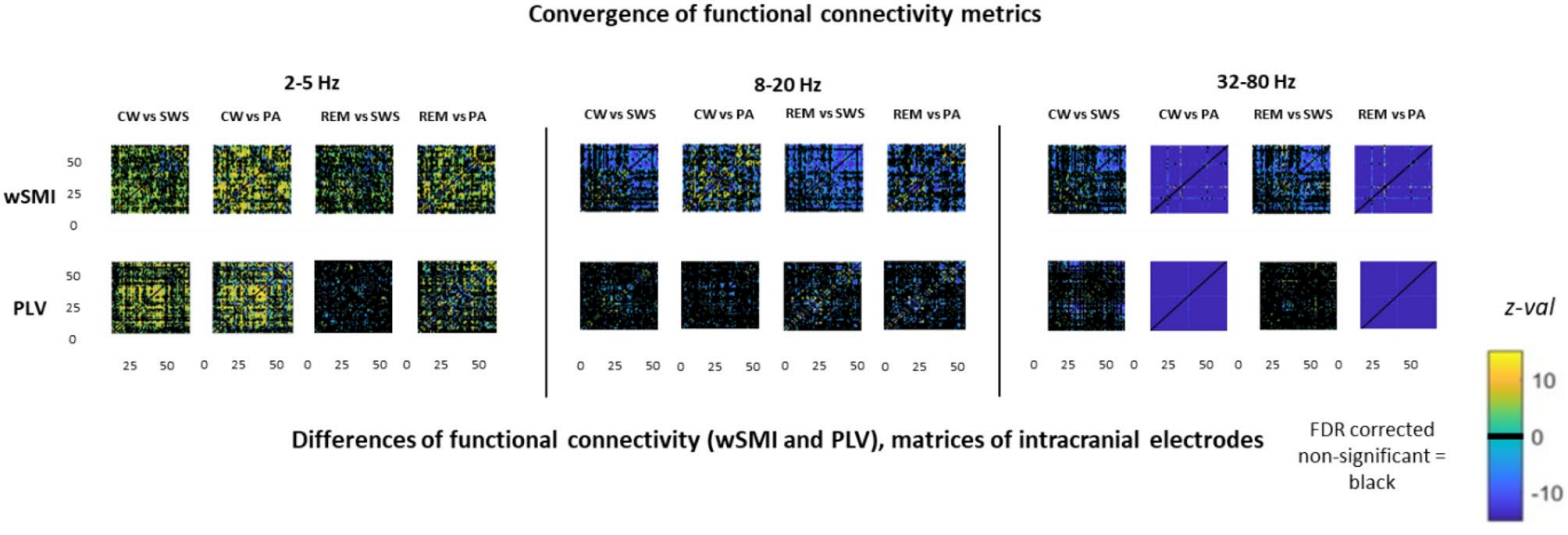
Example functional connectivity matrices estimated by wSMI and PLV (patient 1 of the second experiment). *CW* conscious wakefulness, *SWS* N3 slow wave sleep, *REM* rapid eye movement sleep, *PA* propofol induced general anaesthesia.

While there is growing evidence suggesting that wSMI robustly estimate FC^31^, its use remains recent^20^. To strengthen our findings we thus computed on the same dataset a FC the more traditional Phase Link Value (PLV) to estimate the FC^33^. The main findings have been replicated, in particular in the 2–5 Hz frequency band (see Fig. 4).

**Figure 5:**
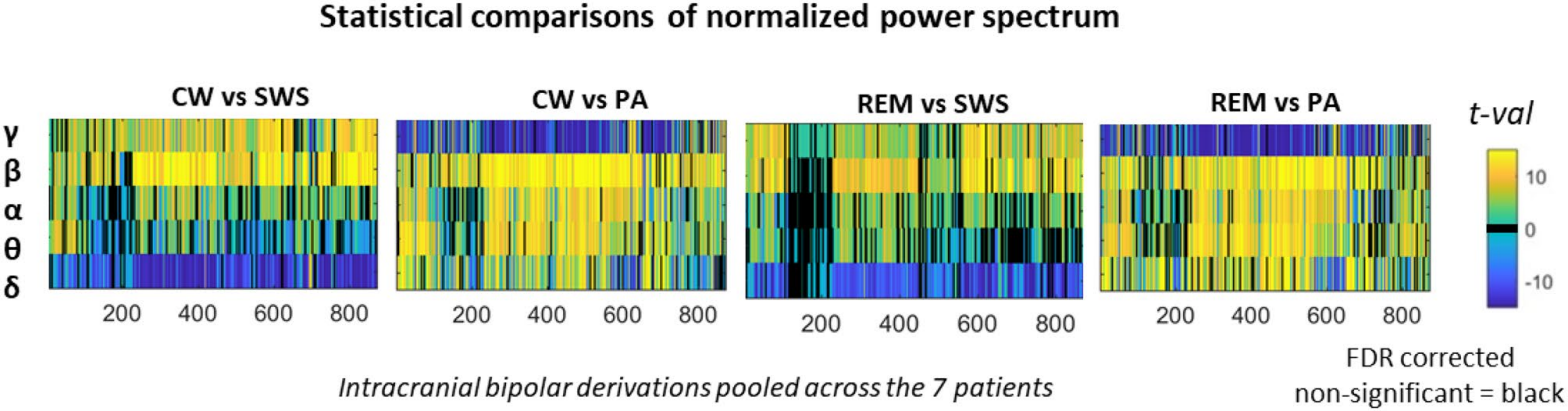
Statistical comparison of power spectrum analysis across the five frequency band of all the bipolar derivation of the patients of the second experiment.

Interestingly, these FC connectivity findings differ from the power spectrum comparison. The power spectrum analysis, performed prior to any other analysis as a data quality check, indeed confirmed the very classical features of scalp EEG analysis in sleep and propofol anaesthesia^15,38–40^ (see Fig. 5). More specifically, concerning the sleep and the wakefulness, the power spectrum analysis is very similar to the recent description reported in the iEEG atlas of normal wakefulness^41^ and sleep^42^ in human provided by the Montreal Neurological Institute (MNI). Whereas the delta band strongly differentiated the SWS (high power spectrum) and the PA (low power spectrum) from both CW and REM, FC in this same frequency band did not. Similarly, while the FC connectivity was higher in the 2–5 Hz frequency band and lower in the 8–20 Hz range, in CW and REM compared to SWS, the power spectrum was classically higher in the theta band and lower in the alpha and beta band during SWS. In all frequency bands but gamma, power spectrums were lower during PA.

### Scalp EEG study—DOC patients

While our previously published data suggested that FC estimated through the wSMI in the 4–10 Hz frequency band was the best electrophysiological signature of conscious states, we failed in the two above experiments to generalize this finding to other modalities of physiological (SWS) and pharmacological (PA) alterations of consciousness. However, we discovered a composite signature (wSMI 2–5 Hz and 8–20 Hz) of conscious state that proved efficient to distinguish CW and REM from SWS and PA.

**Figure 6:**
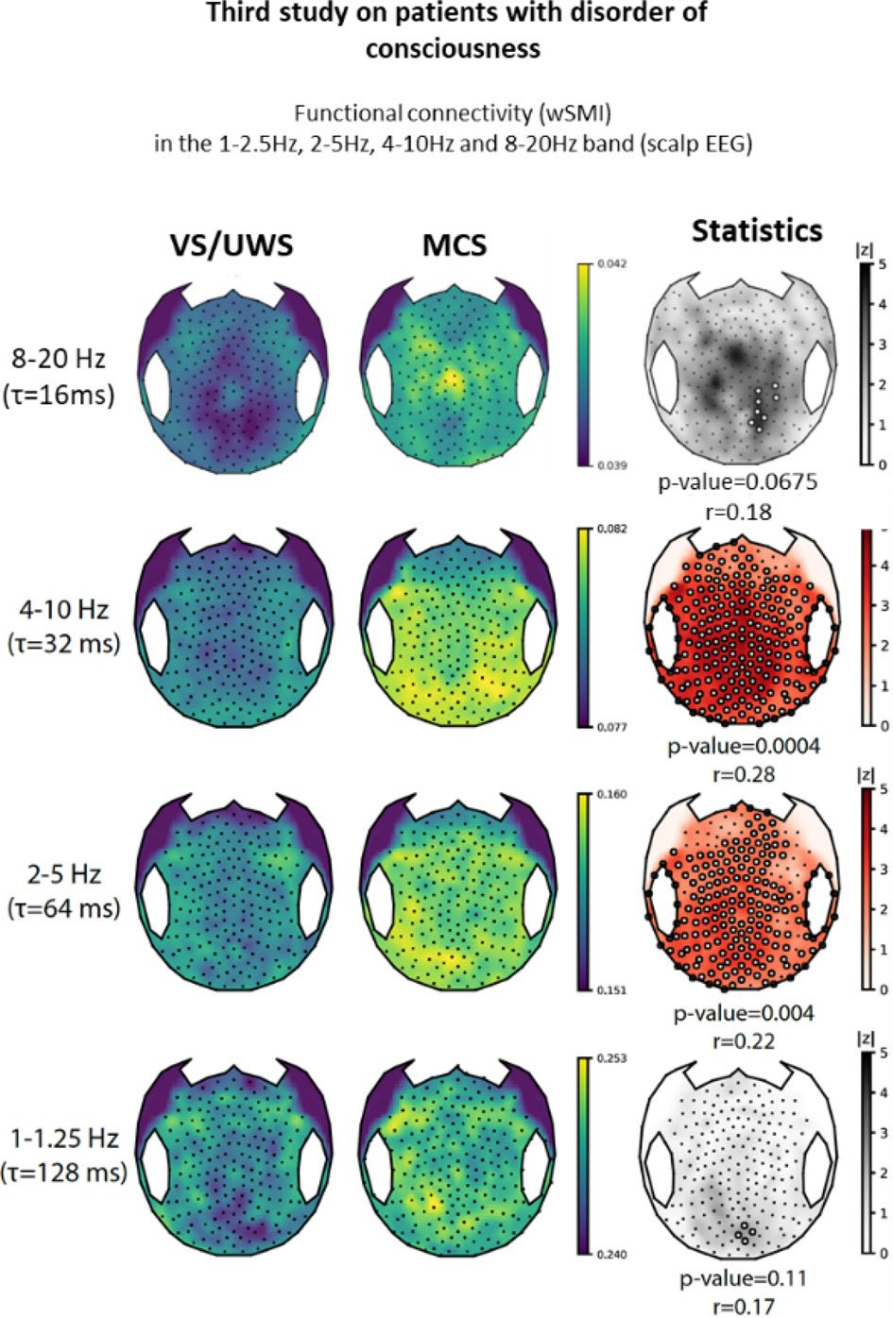
Two-dimensional representation obtained by resuming the value at each electrode by the median value of wSMI between one electrode and all the others for VS/UWS and MCS patients (left columns). This averaging is closely related to the degree measure of a network in graph theory and highlights the sensors that have the strongest connections with other sensors, thus identifying hubs of connections. Results from the permutation cluster-based statistics are represented in the right column. Absolute z-values are plotted with a red color scale when a significant cluster was found and in grey otherwise with the corresponding p-value and effect size r of the cluster. Electrodes belonging to clusters are highlighted by white circles.

In order to probe the generalization of our new measures of conscious state to other alterations of consciousness, we tested if our new signatures could distinguish VS/UWS from MCS in DOC patients. Using 167 five minutes long resting-state high-density EEG recordings acquired from 145 distinct patients (N = 68 VS/UWS and N = 77 MCS), we first showed that wSMI in the 4–10 Hz was able to discriminate VS/UWS from MCS (mean scalp wSMI AUC = 0.66 CI_95_[0.57–0.74], p = 0.0005, effect size r = 0.27 with a significant cluster encompassing almost all scalp electrodes, p = 0.0004), replicating the original findings of King et al. where wSMI was computed during an active paradigm task^20^. Crucially, we then found that wSMI in the 2–5 Hz range was also able to accurately discriminate VS/UWS from MCS (mean scalp wSMI AUC = 0.62 [0.53–0.70], p = 0.01, r = 0.20, with a significant cluster encompassing almost all the scalp, p = 0.004). In contrast, wSMI in the 1–1.25 Hz range did not discriminate these two states, (mean scalp wSMI AUC = 0.54 [0.45–0.62], p = 0.42, r = 0.06 with no significant cluster, p = 0.11). Similarly wSMI computed in the 8–20 Hz range also failed to differentiate these two states (p = 0.42, r = 0.06 with no significant cluster, p = 0.0675) (see Fig. 6). Similar results were found when computing wSMI during the local–global active paradigm as originally described (data not shown).

In addition to the replication of the 4–10 Hz signature previously reported, we noticed that only the 2–5 Hz part of the suspected composite signature of conscious state was thus able to discriminate accurately VS/UWS from MCS. This new signature therefore emerges as a robust signature of conscious states (CW and REM) and regardless of the modality of alteration of consciousness (physiological in SWS, pharmacological in PA, or pathological disorder of consciousness in VS/UWS).

## Discussion

In the present iEEG and EEG study, we first showed that long-range cortico-cortical connectivity measured with the wSMI computed in the theta-alpha band (4–10 Hz),—that was previously shown to be higher for patients in the MCS from those in the VS/UWS, was larger during CW than during SWS. However, and unexpectedly, this measure was inconclusive to discriminate both REM from SWS, and REM and CW from PA. In other terms, FC in the 4–10 Hz does not seem to be a general signature of conscious states. We then discovered that FC computed in a slower band (2–5 Hz)) actually succeeded much better to discriminate correctly conscious states (CW and REM) from the two unconscious states investigated here (SWS and PA). This 2–5 Hz frequency band could correspond to high slow cortical potentials (SCP) band^43–46^. It is worth noticing that the low SCP band was not well explored in our study, as the bandpass filter made it not possible to record oscillations below 0.5 Hz. This measure was found larger in CW and in REM as compared to SWS and to PA in the vast majority of patients. Notably, none of the 12 recorded patients showed a reverse pattern of connectivity between conscious and unconscious states in this high-SCP band. Finally, we could generalize the validity of this new measure in another population of patients and with high-density scalp EEG recordings: wSMI computed in this 2–5 Hz band was significantly larger in the MCS than in the VS/UWS. We showed that wSMI computed in a slower band (1–2.5 Hz) failed to discriminate conscious from unconscious states, both in iEEG and in scalp-EEG, which suggests that there is now bias making the functional connectivity higher in the slower frequencies. Note however that our use of 800 ms long epochs in this scalp EEG study suggests that the right-most range of frequencies of the 2–5 Hz interval contribute the most to this signature of conscious states. A replication with longer epochs could precise this point. Taken together, our results suggest that cortico-cortical connectivity in the high-SCP band could correspond to a general neural signature of consciousness that is valid across a large variety of impairments of consciousness. In addition to enriching cognitive neuroscience of consciousness, the present data and its proposed interpretation may impact clinical practice with a new neurophysiological signature of conscious states.

This proposal is compatible with a core element of the Global Workspace (GW) theory according to which conscious states would correspond to the serial chaining of ~ 200–300 ms discrete states (described in another theoretical frame), each associated with a P3b signature^47^. Indeed, this hypothesis leads to a 3.3–5 Hz frequency range of coherent and synchronized brain-scale patterns that is ideally captured by the wSMI 2–5 Hz. Moreover, our proposal is in line with electrophysiological and fMRI data that highlighted the importance of SCP in cognition and consciousness^10^. He and Raichle listed some explanatory arguments in favor of such a link between SCP (0.1–4 Hz) and conscious states. In particular, they underlined the fact that “long-range cortico-cortical connections terminate preferentially in superficial layers, and thus contribute significantly to SCP”^9,46^.

Our finding of a robust signature of conscious states in high-SCP invites to reconsider the frequency bands involved respectively in conscious access and in conscious states. Indeed, while many studies first reported FC within beta to theta bands as robust signatures of conscious access in the visual and auditory modalities, recent works revealed that SCP plays also a role in conscious access. In two previous SEEG studies, we identified brain-scale increases of connectivity in the alpha–beta band during conscious access to written words^48^, and in the alpha band during conscious access to the regularity auditory series of tones^21,49^. We then identified,—in high-density scalp EEG recordings—, a sharp difference of long-range connectivity within the theta-alpha band between controls in a CW state and patients in the MCS on the one hand, and patients in the VS/UWS on the other hand^20,21^. This progressive slow-down of frequency windows of connectivity when moving from conscious access contrasts, to conscious states contrasts could tentatively suggest mechanistic differences of neural synchronization between these two cognitive processes and states. However, the recent report of SCP signatures of conscious access to visual stimuli complexifies the landscape^43–45^. In this context, the present results further strengthen that conscious states are associated to high-SCP FC. One may further speculate that conscious access,—that appear as a sudden stage related to higher frequencies—, may actually depend on the active maintenance of conscious states and therefore on these high-SCP processes. A dedicated theoretical review on this specific topic could clarify this question by integrating together all solid findings.

Although specific anatomical locations, such as the long distance fronto-parietal mesial structures^20,21,50,51^, have been suggested as essential to the physiology of consciousness, we did not identify reproducible anatomical patterns across patients in terms of functional connectivity. This finding contrasts with the analysis of the power spectrum, which showed important differences between stages, and related to the anatomical localizations of the electrode as in the description recently made in the MNI iEEG atlas of sleep^42^ and wakefulness^41^. These observations tend to corroborate the hypothesis developed in the previous paragraph and the growing number of data suggesting that the neural correlates of conscious states in terms of functional connectivity rely more on the nature and the dynamic of this functional connectivity^8,52,53^ than on its spatial anatomical repartition. Nevertheless, in spite of the lack of clear and reproducible anatomical pattern of functional connectivity, some individual observations could be made. In both Patient #1 of the first experiment and Patient #5 of the second one, the expected effect of an increase of functional connectivity in the 8–20 Hz frequency band during the slow wave sleep was absent for the electrodes of the central region. This could be an indirect consequence of the very specific activity of the central region during the SWS, in which the sleep slow waves are remarkably infrequent^42^, reflecting a possible different position of this region in the network organization during this stage. Patient #2 of the second experiment showed a paradoxical effect especially during the SWS both for the 2–5 Hz and 8–20 Hz frequency bands for a grouping of channels located within the epileptogenic network and the propagation network of this patient. This observation may be interpreted in the same way as the recent observations of elevation of the Phase-Amplitude Coupling during the SWS in the epileptic zone on the interictal recording and independently of sharp epileptic activities^54^. The absence of reproducible anatomical patterns of functional connectivity across the patients could be related to the variability of anatomo-functional organization during conscious states. A recent iEEG study used single-pulse electrical brain stimulations to describe their overall propagation across the different regions of the brain and the different sleep stages. This study suggest that the regulation of conscious awareness and sleep is associated with significant differences in the balance of neural propagation across a wide frontal-parietal network despite the uniform functional connectivity we reported in the present study^55^.

Finally, we will also discuss our findings related to increase of FC in the gamma-band during unconscious states. Recently, loss of consciousness has also been related to hyper-correlated gamma-band activity in anesthetized macaques and sleeping humans through intracranial EEG recordings^37^. We replicated this finding in the present study and generalized it for the first time to anesthetized human subjects. The enhanced inter-dependence of gamma-band activity during alteration of consciousness may reflect suppression of information transfer^56^ consecutive to the well-established decrease of the complexity of electrophysiological signals during unconscious states^19,57–60^. Gamma activity is indeed frequently considered as a macro-scale reflection of a bursty activity pattern observed during sleep and anesthesia^40,61,62^. Massive positive correlations may results from bursty aspecific activities occurring at multiple brain locations. Another interpretation could be that the phase of low-frequency oscillations^63^, changes in cross-frequency interactions between delta and gamma could possibly drive the observed enhanced functional connectivity^37^.

## Materials and methods

### First and second experiment

The first two experiments of this study used a very similar approach. We therefore report their respective experimental procedures in a single section.

#### Patients

All patients had a drug-resistant epilepsy requiring phase II investigation by stereo-electroencephalography. They benefited from this procedure in the department Neurosurgery of Lyon university hospital (Hospital for neurology and neurosurgery Pierre Wertheimer, Lyon University, France). Data, including intracranial-EEG (iEEG) during wakefulness, sleep and general anaesthesia, were collected anonymously and no change the routine management of patients was needed. More specifically the acquisition of intracranial-EEG (iEEG) under general anaesthesia was performed during the surgical implantation and did not modify the duration of the procedure. This study was approved by the local ethics committee (Comité Consultatifs de Protection des Personnes se Prêtant à des Recherches Biomédicales. Authorization No. 22236S). Written informed consent was obtained.

#### Stereo-electroencephalography

Stereo-electroencephalography (SEEG) was performed under general anaesthesia using a frame based Talairach methodology^64^. Electrodes (Microdeep) were manufactured by DIXI (DIXI MEDICAL, 4, chemin de Palente, BP 889, 25025 Besancon, France). Dimensions of each contact are 2 mm in length, 0.8 mm in diameter and the intercontact spacing is 1.5 mm. Each electrode has from 5 to 15 contacts. Signal was recorded by a MICROMED system with frequency sampling of 256 Hz.

SEEG was performed under general anaesthesia using Target-Controlled Infusion (TCI) with propofol (2,6 diisopropylphénol)^65^. Deepness of anaesthesia was controlled by measuring the Bispectral-Index (BIS)^66^.

For the second experiment, the iEEG was recorded (10 min) during the final part of the procedure, during the per-operative imaging control, so before the decrease of anaesthesia and during a perfectly stable level of propofol anaesthesia (PA).

#### Electrodes localisation

All patient had a postoperative MRI in the 24 h following the SEEG. All electrodes were manually localized and their location were put into the MNI space by using MRIcro (Chris Rorden's MRIcro)^67^ and SPM 12.

Tridimensional representation (see Fig. 2a) and matching with functional connectivity matrices were computed by using BrainNet Matlab toolbox (v 1.61, released 20171031)^68^.

#### Sleep staging

Based on American Academy of Sleep Medicine (AASM) recommendation of 2007^69^ and adaptation to iEEG^70^ sleep staging were performed and then controlled by a sleep expert (HB). Periods of 10 min of time, comparable to those obtained under propofol general anaesthesia, were then selected in stable periods of conscious wakefulness (CW), N3 slow wave sleep (SWS) and rapid eye movement sleep (REM). Concerning the N3 slow wave sleep, only periods of stable slow wave activities were selected as we wanted conscious content, as dreaming, to be avoided. Dreaming experiences, so associated with form of consciousness, during N3 slow wave sleep have indeed been associated to fragmentations of the slow activities during this stage^14^.

#### Data pre-processing

A post recording bandpass filter was applied to the data filtering the 50 Hz band (European electrical network frequency), frequency below 0.5 Hz and above 80 Hz, the 256 Hz frequency rate making impossible to make it reliable analysis in higher frequencies. All iEEG data underwent an artefact rejection and a visual analysis using Anywave software (IDDN.FR.001.110001.000.S.P.2014.000.31230 AMU and INSERM)^71^. No channel had to be excluded. All paroxistic or pathological epileptic EEG abnormalities were excluded from the selected recording. The 10 min periods of each stage (wakefulness, N3 slow Wave sleep, rapid eyes movement sleep and propofol induced general anaesthesia) was divided into 10 s periods of time. A re-referencement of electrode contacts to a bipolar montage with their nearest neighbour on the same physical electrode. Specifically, before functional connectivity analysis, non-contiguous electrode contacts were excluded to avoid massive biases.

#### Power spectrum analysis

The power spectrum analysis of each frequency band (δ: [0.5–4 Hz[; θ: [4–8 Hz[; α: [8–12 Hz[; β: [12–30 Hz[; γ [30–70 Hz]) was performed with Matlab using the Fieldtrip toolbox (Donders Institute for Brain, Cognition and Behaviour, Radboud University, The Netherlands)^72^. A multitaper methods and a path of time of 0.5 s was used. Each frequency band and each stage were normalized by computing the sum of power in a frequency band reported to the power on all frequency bands of the spectrum sum.

#### Functional connectivity analysis

Functional connectivity was estimated by computing the weighted symbolic mutual information index (wSMI) previously described by King et al.^20^ as a relevant measure to discriminate between different states of consciousness. Conversely to phase or amplitude correlation measures, this index, derived from the permutation entropy analysis, can detect non-linear coupling between pair of bipolar derivation. First, signal was reduced into a limited set of discrete symbols, made of groups of sub-vectors, including a certain number of points (k = 3, so 6 existing patterns) sampled with a particular temporal interval. This temporal interval, τ, determined the frequency range for which the index become sensible. Every sub-vector corresponds to a particular symbol, assigned according to different reciprocal patterns that the three points can assume^32^. Here the frequency bands investigated were 32–80 Hz (τ = 4 ms), 16–40 Hz (τ = 8 ms), 8–20 Hz (τ = 16 ms), 4–10 Hz (τ = 32 ms), 2–5 Hz (τ = 64 ms) and 1–2.5 Hz (τ = 128 ms). The symbols’ probability density and counting their mutual occurrence over two time-series the index was determined prior to compute an estimate of the coupling between each pair of electrodes^20,32^: (x and y being the pairs of symbols of the two time series). To compute wSMI, the SMI is weighted to disregard conjunctions of identical or opposite-sign symbols: the weights were set to zero for identical symbols in the two signals, which could be elicited by a unique common source, and for opposed symbols, which could reflect the two sides of a single electric dipole^20^.

In addition to wSMI measurement, we performed an analysis based on rhythmic synchronization in order to provide another modality of functional connectivity measurement using classic tool. We used a Phase Locking Value (PLV) computed with the Fieldtrip tool box of Matlab R2017a v9.2.0.556344^33,72^.

#### Statistical analysis

All statistical analysis was performed with Matlab R2017a v9.2.0.556344 and the stats toolbox (Copyright 1984–2017, THE MATHWORKS, Natick, Massachusetts, USA). A Welch’s test without hypothesis of equal variances was used to perform the statistical comparisons on the normalized power spectrum between the different stages across the 5 frequency bands. A non-parametric two-tail Mann–Whitney U-test was used for comparisons between wSMI values of each state of consciousness. The area under the curve (AUC) of the receiver operating characteristic curve (ROC) was then reported. Note that AUC is related to the U statistic of the Mann–Whitney U test: $AUC=\frac{U}{n1*n2}$ (where n1 and n2 are the size of both groups). Control for multiple comparisons was made by using the False Discovery Rate (FDR)^73–75^. The 0.05 threshold of significance was adapted at each test according to this adjustment with the FDR.

Comparison of the different mean wSMI for each frequency band between the stages for each patient was performed by using a student t-test.

### Third experiment

#### Population

Five minutes resting state EEG were acquired from a convenience sample of patients suffering from disorders of consciousness (DoC) assessed in la Pitié-Salpêtrière university hospital (Paris, France) from February 2014 to July 2018. Patients were assessed by trained physicians using the dedicated Coma Recovery Scale revised^76,77^. This specialized scale not only quantifies behavior responses to a set of predetermined and hierarchical items in six different domains (auditory, visual, motor, oro-motor, communication and arousal), but also determines the consciousness state of the patient according to some key behaviors elicited by the passation of the scale. Patients were thus diagnosed as being in a vegetative state/unresponsive wakefulness syndrome (VS/UWS) or in a minimally conscious state (MCS). A total of 203 recordings were acquired from 167 unique DoC patients. After the automated preprocessing pipeline (see below), 167 valid recordings acquired from 145 unique patients were amenable for analysis. The population, 68 VS/UWS and 77 MCS patients, was typical of DoC patients, with 87 males and 54 females (sex-ratio = 1.61), median age of 47.4 IQR [30.4–62.6] years. Etiologies were anoxia in 37%, traumatic brain injury in 24%), stroke in 5% and other causes in 34%. Median delay from injury was 58 [31.0–184.0] days. Consent was obtained from the patient’s relative. The protocol conformed to the Declaration of Helsinki, to the French regulations, and was approved by the local ethic committee (Comité de Protection des Personnes; CPP n° 2013-A01385-40) Ile de France 1 (Paris, France). Written informed consent was obtained.

#### EEG acquisition and preprocessing

Five minutes resting state scalp EEG were acquired in a quiet room while the patients received no particular instruction. Recordings were done using a NetAmps 300 Amplifier (ELECTRICAL GEODESICS, Eugene, Oregon) with a high-density sponge-based 256 channels HydroCel Geodesic Sensor Net (ELECTRICAL GEODESICS, Eugene, Oregon) referenced to the vertex at 250 Hz sampling frequency. Impedances were checked before the beginning of the recording and set below 100 kΩ.

An automatized and hierarchical preprocessing workflow written in Python, C, bash shell scripts and based on open source technologies, including the software MNE^78^, was used for artefact removal and quality assessment^21,36,79^. Shortly, this previously described pipeline followed the subsequent steps: (1) Filtering: raw data were band-pass filtered (0.5 Hz 6th-order Butterworth high-pass filter and 45 Hz 8th-order Butterworth low-pass filter) with 50 Hz and 100 Hz notch filters. (2) Epoching: filtered data were cut into 800 ms epochs with a 550 to 850 ms random jitter in-between (these timings were chose to reproduce the original wSMI analysis in which wSMI was computed on the 800 ms baseline periode of an active auditory oddball paradigm^20^). (3) Bad channels and bad epochs removal: channels that exceeded a 100 μv peak-to-peak amplitude in more than 50% of the epochs were rejected. Channels that exceeded a z-score of 4 across all the channels mean variance were rejected. This step was repeated two times. Epochs that exceeded a 100 μv peak-to-peak amplitude in more than 10% of the channels were rejected. Channels that exceeded a z-score of 4 across all the channels mean variance (filtered with a high pass of 25 Hz) were rejected. This step was repeated two times. The remaining epochs were digitally transformed to an average reference. Rejected channels were interpolated. Finally, EEG were deemed to pass this preprocessing step if at least 70% of the channels and at least 30% of the epochs were kept.

#### Functional connectivity analysis

As previously described, wSMI was computed on the 800 ms epochs using a k = 3 kernel in the different frequency bands, respectively: tau = 32 ms (4–10 Hz), tau = 64 ms (2–5 Hz) and tau = 128 ms (1–1.25 Hz), yielding a single value of wSMI in each frequency band for each pair of scalp electrodes (224X(224–1)/2 = 24,976) per epochs and per patient. A single value for each electrode was then obtained by computing the median value of the connectivity between this electrode and every other scalp electrode, resulting in a measure related to the degree of a network in graph theory analysis. These median values were then averaged over time using the trimmed mean 80%, a robust estimator of central tendency^80^. A single two-dimensional topography of the 224 scalp electrodes was thus characterizing each patient. Furthermore, the mean wSMI over the scalp was also used as a measure of the overall magnitude of functional connectivity in each frequency band.

#### Statistical analysis

We compared the wSMI in each frequency band between VS/UWS and MCS, both at the level of the mean wSMI over the scalp (wSMI_mean_) and at the topography level. Populations were compared using the non-parametric Mann–Whitney U test. The discriminative power of wSMI_mean_ to distinguish VS/UWS from MCS patients was assessed using the area under the ROC curve (AUC) and its bootstrapped 95% confidence interval computed using 10,000 iterations. Note that AUC is related to the U statistic of the Mann–Whitney U test:

$$AUC=\frac{U}{n1*n2}$$

where n1 and n2 are the size of both groups.

For the topography analysis, we used a cluster-based permutation procedure^81,82^ to control for multiple comparisons at sensor level. The first step of this procedure consisted of the comparison of the wSMI values at each 224 sensors, using the same statistical test as previously described. Spatial clusters of z-statistic corresponding to type I error of 5% were then constructed using the neighboring matrix of electrodes. Each resulting cluster were assigned a value, known as the cluster mass, which corresponds to the sum of the z-statistic of the individual components of the cluster. Random permutation (N = 10,000) of the patient’s label were then used to create a surrogate distribution of the cluster masses under the null hypothesis. One can then compute the probability of each cluster constructed with the original data to be observed under the null hypothesis. In addition to p-values, Effect sizes were reported using the measure *r*:

$$r=\frac{z}{\sqrt{N}}$$

where *z* is the z-statistic of the Mann–Whitney U test and *N* the size of the population.

This effect size measure was computed both at the level of the wSMI_mean_, but also at the level of the cluster (effect size computed on the mean over electrodes belonging to the cluster).

### Ethical statements

We confirm that we have read the Journal’s position on issues involved in ethical publication and affirm that this report is consistent with those guidelines.
